# Bones and adrenal organogenesis: how embryonic osteocalcin influences lifelong adrenal function

**DOI:** 10.1172/JCI157200

**Published:** 2022-02-15

**Authors:** Typhanie Dumontet, Gary D. Hammer

**Affiliations:** University of Michigan, Ann Arbor, Michigan, USA.

## Abstract

Osteocalcin is a hormone produced in bones by osteoblasts during bone formation. Numerous studies have demonstrated that adrenal gland–derived glucocorticoids inhibit osteocalcin production, which can ultimately cause deleterious bones loss. This loss establishes a unidirectional endocrine relationship between the adrenal glands and bone, however, whether osteocalcin reciprocally regulates glucocorticoid secretion remains unclear. In this issue of the *JCI*, Yadav and colleagues address how bone-derived osteocalcin influences adrenal organogenesis and function. Using a large variety of animal models, the authors established that embryonic osteocalcin signaling, specifically through the GPR158 receptor, regulates postnatal adrenal steroid concentrations throughout life. This work has translational potential, and we await future investigations that determine whether modulating osteocalcin levels could promote endogenous adrenocortical function in adrenocortical hypoplasia and glucocorticoid deficiency.

## Multiple organs influence adrenal function

The adrenal glands are essential organs involved in body homeostasis and the response to stress by producing critical hormones and neurotransmitters. The outer cortex of the gland is specialized in producing steroid hormones, such as glucocorticoids that are synthesized in response to activation of the hypothalamic/pituitary/adrenal axis (HPA) and mineralocorticoids controlled by the renin-angiotensin-aldosterone system (RAAS). Given the critical importance of adrenal hormones in regulating numerous key physiological processes (metabolism, blood pressure, immunity, behavior, and reproduction), it is not surprising that a variety of organs reciprocally influence adrenal function and hence define a classic (albeit secondary to HPA and RAAS control) endocrine feedforward and feedback loop. The influence of the gonads on adrenal function was originally described in the 1930s, when researchers noted that female adrenal glands were heavier than those of males, suggesting a potential role for sexual hormones in regulating adrenal gland growth (1). This role was further substantiated by early observations that adrenal cortex stem cell capacity is sexually dimorphic, the cellular and molecular basis of which has been recently unraveled (2–5). This information has important implications for adrenal diseases, since androgens protect from the development of adrenal hyperplasia and hypersecretion syndrome in mouse models (4, 6). Early studies in mice have also shown that administration of thyroid extracts leads to hypertrophy of the inner adrenal cortex (7), observations that were recently further investigated by Huang and colleagues. Huang and co-authors described a population of cells localized in the innermost part of the cortex, expressing the receptor *Thrb*β (8). Studies performed in mice showed that triiodothyronine (T3) treatment triggers transcriptional changes associated with the stimulation of enzymes and key regulators of the cholesterol synthesis pathway (9). While the findings imply that hormonal synthesis may be affected, the relationship between thyroid and adrenal function is largely irresolute, and the clinical implications remain to be explored. In this Commentary, we offer a basic understanding of the article published in this issue of the *JCI* by Yadav et al. (10), which provides a follow-up to previous studies that have assessed various endocrine roles of osteocalcin (11,12).

## Osteocalcin is a bone-produced hormone

Mammalian bone is a nanocomposite composed of calcium phosphate (hydroxyapatite) mineral embedded in type 1 collagen. This biomaterial property affords the skeleton resilient structural support for muscle-driven locomotion, while simultaneously serving as a hormone-responsive reservoir for calcium and phosphate ions. In addition to bone’s established role in the regulation of mineral metabolism, an increasing body of evidence suggests that skeletal cells also participate in balancing global energy homeostasis by producing circulating factors that function in an endocrine fashion. For example, osteocalcin (*BGLAP*), a protein produced exclusively by osteoblasts, has been shown to regulate glucose homeostasis by promoting insulin secretion by the pancreas (13). Subsequent studies in osteocalcin-null mice (*Ocn^–/–^*) by the Karsenty group suggested additional endocrine functions for osteocalcin in behavior and reproduction that manifest by activation of distinct GPCRs in brain and testis (11, 12, 14, 15).

The existence of a functional relationship between the adrenal gland and bone was originally described in the 1930s in patients with glucocorticoid excess and bone loss (osteoporosis) accompanied by an increased risk of fractures (16). Studies have previously shown that osteocalcin is transcriptionally repressed by glucocorticoids via the receptor GR (17). To our knowledge, the hypothesis that bones reciprocally regulate adrenal function has never been tested, despite the well-known role of glucocorticoids in bone architecture. Here, Yadav and colleagues hypothesized that bones and adrenal glands constitute a classical endocrine system composed of both a bone-to-adrenal feedforward activation arm and an adrenal-to-bone feedback inhibition arm (10). Through the implementation of multiple mouse models that included genetic loss (14) and gain-of-function of osteocalcin (11) and its receptor (18), and experiments in primates, the authors assessed the role of osteocalcin in adrenal function and homeostasis (10).

## Osteocalcin stimulates corticosterone and aldosterone production through GPR158

Following injection of osteocalcin into adult mice and macaques, Yadav and researchers observed that blood levels of corticosteroid hormones rapidly increased. mRNA levels of the adrenal cortex enzymes *Cyp11b2* and *Cyp11b1* were also upregulated, supporting a transcriptional effect on adrenal steroidogenic enzymes. Similar results were observed with *Mc2r* and *Agtr1a/b* expression, suggesting that osteocalcin may potentiate adrenocorticotropic hormone (ACTH) and angiotensin signaling. To identify the adrenal receptor for osteocalcin, the authors performed quantitative PCR (qPCR) analysis and used RNAscope technology to demonstrate that *Gpr158* was expressed throughout the cortex. They showed that corticosteroid levels were decreased in a *Gpr158* loss-of-function mouse model and that osteocalcin injections failed to rescue the endocrine phenotype. This result suggests that GPR158 is the main receptor by which osteocalcin stimulates adrenal function (10).

## Developmental osteocalcin is essential for adult adrenal function

Despite the convergent pieces of evidence indicating the role of osteocalcin signaling in adrenal steroidogenesis, the authors were surprised to find that osteocalcin-deficient mice had normal adrenal hormone levels compared with their nondeficient littermates. However, osteocalcin-deficient mice born from osteocalcin-deficient mothers had lower adrenocortical hormone levels and decreased expression of steroidogenic enzymes in the adult adrenal cortex. Yadav and authors hypothesized that maternal and/or embryologic osteocalcin might regulate steroidogenesis postnatally. Through a combination of various breeding schemes, the authors showed that embryonic exposure to osteocalcin influenced postnatal corticosteroid levels. Not only were adrenal hormonal levels dampened in adults, but physiological functions regulated by adrenal steroids were concomitantly hampered. Blood pressure and circulating potassium concentrations were lower in mice lacking GPR158 in adrenocortical cells and in osteocalcin-deficient mice born from osteocalcin-deficient mothers. Besides the conventional ACTH stimulation test, the authors assessed the acute stress response by using short exposure to 2,3,5-trimethyl-3-thiazoline (TMT), a predator odor known to induce intense stress in rodents. They observed a dampened response in corticosterone induction after exposure. Consistently, none of these functions was affected in osteocalcin-deficient adult mice that received maternal osteocalcin during development, suggesting rescued adrenal function (10).

To better understand how embryonic osteocalcin signaling could influence adrenal steroidogenesis later in adult offspring, the authors hypothesized that osteocalcin may promote cell proliferation during adrenal organogenesis. By performing immunohistochemistry for Ki-67, the authors demonstrated a deficiency in cell proliferation in adrenal cortex of embryos lacking GPR158 and in osteocalcin-deficient mice born from osteocalcin-deficient mothers. These observations correlated with an adrenal growth delay that manifested after E14.5 and eventually led to smaller adrenal glands at E16.5. Conversely, the adrenal glands were larger in an osteocalcin gain-of-function mouse model. These results support the hypothesis that osteocalcin signaling during development contributes to adrenal gland growth, with effects sustained throughout adult life. At the molecular level, decreased expression of *Sf1* (also known as *Nr5a1*) and its target genes *Cyp11b2* and *Cyp11b1* suggests that osteocalcin signaling mediates its effects on the adrenal cortex in part through an unknown mechanism of regulation involving critical adrenal transcriptional programs (10).

## Osteocalcin rescues adrenal insufficiency of *Mc2r^–/–^* mice

Since the adrenal cortex is functionally regulated by ACTH, the authors raised the question of whether osteocalcin promotes adrenal growth and steroidogenesis independently of regulation by the HPA axis. *Mc2r*, which encodes the ACTH receptor, is essential for transducing ACTH signaling, thereby contributing to the maintenance of adrenal differentiation and glucocorticoid production. *MC2R* mutations have been responsible for familial glucocorticoid deficiency (19), and *Mc2r^–/–^* mice mostly mimic the human phenotype, consisting of adrenal hypoplasia and insufficiency (20). Interestingly, adrenal glands of E18.5 *Mc2r^–/–^* embryos carried by osteocalcin-injected mothers were larger when compared with those of vehicle-injected embryos. This result was also accompanied by a partial rescue of *Mc2r^–/–^* pup lethality upon delivery, suggesting that osteocalcin functions either independently of ACTH signaling or downstream of the ACTH receptor to mediate its effects on adrenal growth and differentiation (10).

## Conclusions

**Figure 1:**
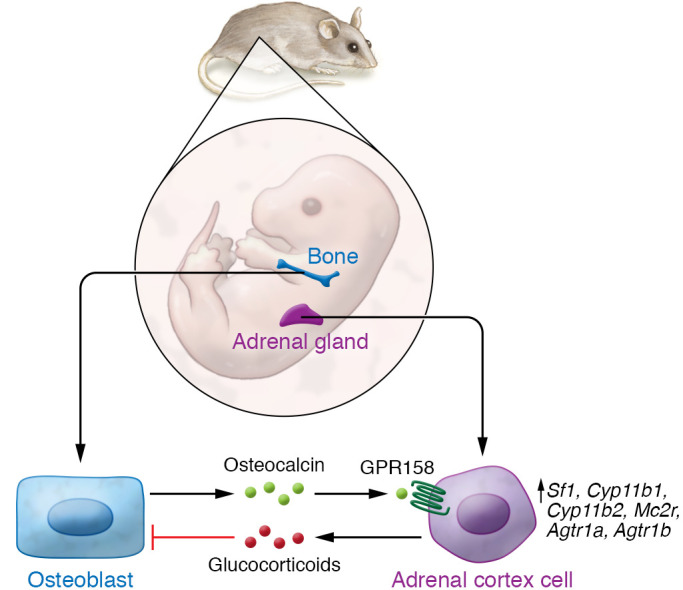
The bone-adrenal endocrine circuit. Yadav and colleagues detailed how bone-derived osteocalcin, produced by osteoblasts and released into the bloodstream, acts distantly on adrenal cortex cells. Through activation of GPR158, osteocalcin promotes adrenal cortex proliferation and differentiation during embryogenesis, in part via upregulation of the key steroidogenic genes *Sf1* (also known as *Nr5a1*), *Cyp11b1*, *Cyp11b2*, *Mc2r*, *Agtr1a*, and *Agtr1b* (10). These results complement previously described glucocorticoid effects, thus completing the bone-adrenal endocrine circuit.

Ninety years after Dr. Cushing recognized that osteoporosis was a common deleterious effect of glucocorticoid excess on bone (16), Yadav and colleagues define the complementary other half of a bone-adrenal endocrine circuit (Figure 1). They detail the feed-forward activation by which bone-derived osteocalcin modulates fetal adrenal homeostasis, with physiological consequences still imprinted throughout adult life. Whether or to what extent the adrenal effects of osteocalcin described in the mouse are conserved in humans will require additional studies, particularly in light of recent findings in the recent osteocalcin-deficient mouse models (21–23), which failed to replicate the metabolic phenotypes reported by Karsenty’s group. It should be noted that the current results obtained in macaques, together with the demonstration that osteocalcin injection can partially rescue adrenocortical hypoplasia and glucocorticoid deficiency in *Mc2r^–/–^* mice, lend support for the human translation of the bone/adrenal axis. The clinical implications of adrenal failure following long-term glucocorticoid treatment warrant additional studies to determine whether modulation of osteocalcin levels restores endogenous adrenocortical function and, if so, might be developed to treat adrenal insufficiency. It will also be important to determine whether polymorphisms in *BGLAP* are associated with impaired adrenal function. Such lines of investigation will clarify the biology of the bone-adrenal endocrine system reported by Yadav et al. (10) and can be expected to fuel discoveries with physiologic, pathophysiologic, and therapeutic implications for years to come.

## Version 1 02/15/2022

Electronic publication
